# A Hybrid Artificial Intelligence Approach for Down Syndrome Risk Prediction in First Trimester Screening

**DOI:** 10.3390/diagnostics15121444

**Published:** 2025-06-06

**Authors:** Emre Yalçın, Serpil Aslan, Mesut Toğaçar, Süleyman Cansun Demir

**Affiliations:** 1 Department of Obstetrics and Gynecology, Division of Perinatology, Cukurova University School of Medicine, 01330 Adana, Turkey; dremreyalcin23@gmail.com (E.Y.); cansundemir@gmail.com (S.C.D.); 2 Department of Computer Engineering, Faculty of Engineering and Natural Sciences, Malatya Turgut Ozal University, 44210 Malatya, Turkey; 3 Department of Management Information Systems, Faculty of Economics and Administrative Sciences, Fırat University, 23119 Elazig, Turkey; mtogacar@firat.edu.tr

**Keywords:** Down syndrome, first trimester prenatal screening, artificial intelligence, risk assessment, feature extraction

## Abstract

**Background/Objectives:** The aim of this study is to develop a hybrid artificial intelligence (AI) approach to improve the accuracy, efficiency, and reliability of Down Syndrome (DS) risk prediction during first trimester prenatal screening. The proposed method transforms one-dimensional (1D) patient data—including features such as nuchal translucency (NT), human chorionic gonadotropin (hCG), and pregnancy-associated plasma protein A (PAPP-A)—into two-dimensional (2D) Aztec barcode images, enabling advanced feature extraction using transformer-based deep learning models. **Methods**: The dataset consists of 958 anonymous patient records. Each record includes four first trimester screening markers, hCG, PAPP-A, and NT, expressed as multiples of the median. The DS risk outcome was categorized into three classes: high, medium, and low. Three transformer architectures—DeiT3, MaxViT, and Swin—are employed to extract high-level features from the generated barcodes. The extracted features are combined into a unified set, and dimensionality reduction is performed using two feature selection techniques: minimum Redundancy Maximum Relevance (mRMR) and RelieF. Intersecting features from both selectors are retained to form a compact and informative feature subset. The final features are classified using machine learning algorithms, including Bagged Trees and Naive Bayes. **Results**: The proposed approach achieved up to 100% classification accuracy using the Naive Bayes classifier with 1250 features selected by RelieF and 527 intersecting features from mRMR. By selecting a smaller but more informative subset of features, the system significantly reduced hardware and processing demands while maintaining strong predictive performance. **Conclusions**: The results suggest that the proposed hybrid AI method offers a promising and resource-efficient solution for DS risk assessment in first trimester screening. However, further comparative studies are recommended to validate its performance in broader clinical contexts.

## 1. Introduction

Down Syndrome (DS) is the most common chromosomal abnormality observed at birth, with an incidence rate of about 1 in 1000. This rate increases with maternal age. DS is primarily caused by the presence of an extra copy or partial duplication of the long arm of chromosome 21. This chromosomal abnormality typically occurs due to nondisjunction during meiosis II; however, translocation and mosaicism are also responsible for a smaller number of cases. Clinically, DS is characterized by distinctive facial features, congenital heart defects, and varying degrees of cognitive impairment. These characteristics are largely associated with the q21.1, q22.2, and q22.3 regions of chromosome 21 [1].

A key objective of prenatal care is the early detection of fetal anomalies to enable timely medical intervention during pregnancy or shortly after birth. Although parents have the right to know their unborn child’s health status, this right is limited by the available diagnostic technologies and current medical knowledge [2,3]. Because many congenital conditions can only be diagnosed once structural or functional abnormalities reach detectable thresholds, the development of accurate, early screening tools is essential.

Genetic disorders such as DS carry significant social, psychological, and economic burdens. While these conditions are not curable, early detection enables better preparation, counseling, and medical planning. In this context, non-invasive screening methods have become more important. One such method is the first trimester combined screening test, which uses maternal serum markers—free β-human chorionic gonadotropin (β-hCG) and pregnancy-associated plasma protein A (PAPP-A)—along with an ultrasonographic measurement of nuchal translucency (NT). Performed between the 11th and 14th weeks of gestation, this test offers detection rates of up to 90% for DS and other chromosomal anomalies, such as trisomy 13, trisomy 18, and Turner syndrome [4,5,6,7].

In pregnancies affected by DS, PAPP-A levels are typically lower and β-hCG levels are higher than expected. These markers are interpreted using “multiple of the median” (MoM) values, which normalize for gestational age [8,9]. Additional risk indicators include NT thickness greater than 2.5 mm, pregnancy-associated plasma protein A (PAPP-A) less than 0.4 MoM, beta-human chorionic gonadotropin (β-hCG) greater than 2.5 MoM, and absence of the nasal bone. Further diagnostic evaluation, such as fetal echocardiography and cell-free DNA (cfDNA) testing, is recommended when NT measurements are ≥3 mm or above the 99th percentile [10]. Based on these parameters, risk levels are categorized as high (>1/250), moderate (1/250–1/1000), or low (<1/1000) [11]. Although screening provides valuable risk estimates, a definitive diagnosis still requires invasive procedures, such as chorionic villus sampling (CVS), amniocentesis (AS), or cordocentesis (CS). These methods carry risks, including miscarriage, infection, and membrane rupture. Consequently, many patients hesitate to undergo invasive testing unless it is absolutely necessary. Furthermore, concerns over malpractice may cause healthcare providers to recommend these procedures based solely on screening results, even when ultrasonography reveals no abnormalities. This underscores the need for more reliable, non-invasive tools.

Recent advancements in artificial intelligence (AI) have opened new possibilities in prenatal diagnostics [12]. In countries like China, hundreds of laboratories now apply AI-based statistical models—often combined with ultrasound data—to assess DS risk during the first trimester [13,14,15]. Machine learning (ML) algorithms are increasingly being used to interpret large-scale screening data and provide individualized risk predictions. Several studies have demonstrated the potential of AI in DS risk estimation. These include approaches based on support vector machines (SVMs), Bayesian classifiers, neural networks, and ensemble learning techniques applied to maternal serum markers and fetal ultrasound measurements [16,17,18,19,20,21,22]. Despite these developments, there is still a need for integrated, high-performance models that combine advanced machine learning techniques with clinical screening protocols.

### 1.1. Related Work

The following are findings from studies similar to the subject of this article: Nguyen-Hoang et al. [23] investigated the ability of AI and ML algorithms to predict pre-eclampsia risk in the first three months of pregnancy in a large pregnancy cohort in Asia. The study found that the AI + ML model developed after calibrating biochemical analysis devices demonstrated higher accuracy in detecting pre-eclampsia compared to the fetal medicine foundation risk prediction model. Verma et al. [24] developed an algorithm called adaptive stochastic gradient descent (ASGIA) to detect structural abnormalities in fetuses and identify potential risks. According to the study’s findings, fetal developmental abnormalities could be classified with high accuracy using second-trimester ultrasound scans supported by advanced technology. The results achieved a remarkable success rate of 98.64%. Alonso et al. [25] developed a new screening method to identify women at risk for DS during the first three months of pregnancy. To this end, they trained different ML techniques using a dataset consisting of 90,532 individuals, with less than 1% having a positive case. The models analyzed demographic factors, such as the mother’s age, weight, and ethnic origin; and biochemical parameters, such as nuchal translucency, PAPP-A, and B-HCG levels. The results showed that the ROC-AUC values ranged from 0.970 to 0.982, with sensitivity and specificity rates reaching 94%. Chen et al. [26] evaluated multiple ML algorithms to predict the risk of DS in the first trimester of pregnancy among women of East Asian origin. The study utilized a dataset comprising a total of 14 variables, including maternal age, nuchal translucency thickness, and various serum biomarkers. Among the 12 different algorithms applied, the highest sensitivity rate (84%) was achieved with LightGBM, while the highest AUC score (0.939) was obtained using an artificial neural network (ANN) and a long short-term memory (LSTM)-based model.

### 1.2. Research Question and Contributions

This study seeks to answer the following question: Does converting one-dimensional (1D) patient data into two-dimensional (2D) barcode images and applying transformer-based feature extraction improve the accuracy of DS risk assessment systems? This study introduces a novel, hybrid AI framework designed to improve the prediction of DS risk levels during the first trimester of prenatal screening. The key contributions of this research are summarized below:

- We convert 1D prenatal screening data into 2D Aztec barcode images to improve feature extraction using transformer models.
- We combine multiple transformer architectures (DeiT3, MaxViT, and Swin) to enrich feature representation.
- A dual feature selection process (mRMR and RelieF) reduces dimensionality while maintaining accuracy.
- Provides a practical AI-based tool for reliable and efficient DS risk prediction in the first trimester.

The remainder of this article is organized as follows: Section 2 describes the approaches used in the material and proposed model. Section 3 presents the experimental analysis and results. Section 4 discusses the findings and limitations. Finally, Section 5 summarizes the study’s conclusion and outlines future work.

## 2. Materials and Methods

### 2.1. Dataset

In order to be used in this study, data from the combined double-screening test applied to 958 women who had a singleton pregnancy in the first trimester between 2020 and 2024 at the Gynecology and Obstetrics Unit of Çukurova University (Adana, Turkey) were collected. The research ethics committee of Çukurova University Faculty of Medicine approved the purpose of the study and the data collection process. Patient records and data were collected from the hospital’s gynecology and obstetrics clinic and biochemistry laboratories. Since the patient data were anonymized, no patient identification information was used. DS (Trisomy 21) was diagnosed in 18 of the 958 unique pregnancies included in the study dataset. In addition, four cases with other chromosomal abnormalities, such as Trisomy 18 and Trisomy 13, were identified. The remaining 936 pregnancies, which were considered chromosomally normal, were evaluated as the control group.

The features in the dataset used in the study are given in Table 1. Four features, whose descriptions and data types are given in Table 1, were used as markers that could help infer the risk of DS: “hCH-MoM”, “PAPP-A MoM”, “NT”, and “NT MoM”. The “Down Risk” indicator was used to estimate the categorical data. Prenatal screening reports use the features’ standard deviations or medians to determine the DS risk [27].

**Figure 1:**
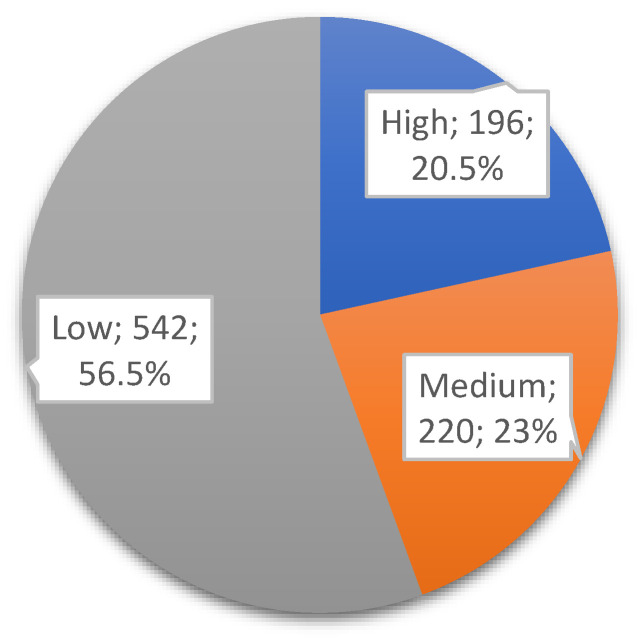
Distribution of Down Risk attribute of dataset.

Figure 1 represents the class distributions of the DOWN target variable in the dataset used. After applying the clinically accepted risk thresholds (high: <1:250; medium: 1:250–1:1000; low: >1:1000), the dataset was categorized into three risk groups. Among the 958 records, 196 (~20.5%) were labeled as high risk, 220 (~23%) as medium risk, and 542 (~56.5%) as low risk, as shown in Figure 1.

The sample size used in the study was determined by considering the effect sizes observed in previous, similar studies, as well as statistical power analyses. The primary endpoint is an evaluation of how accurately the model predicts DS risk.

According to standard first trimester prenatal screening (FTS) protocols, DS risk is classified as high, moderate, or low. Those classified as high risk (below the 1:250 threshold) were referred for invasive diagnostic methods, such as amniocentesis or CVS, in accordance with the relevant clinical guidelines. Where such invasive tests were performed, the diagnosis was confirmed through fetal karyotyping. Additionally, postnatal karyotype analysis has been utilized in appropriate cases to enhance diagnostic accuracy. In this context, the classification labels in the dataset are based not only on screening findings, but also on confirmatory clinical diagnoses, particularly in the high-risk group. In particular, the ground truth labels used to train the model for high-risk cases were validated using confirmatory diagnostic procedures, such as amniocentesis or postnatal karyotyping, to ensure clinical validity.

### 2.2. Barcode Recognition and 2D Data Generation with Aztec Technique

**Figure 2:**
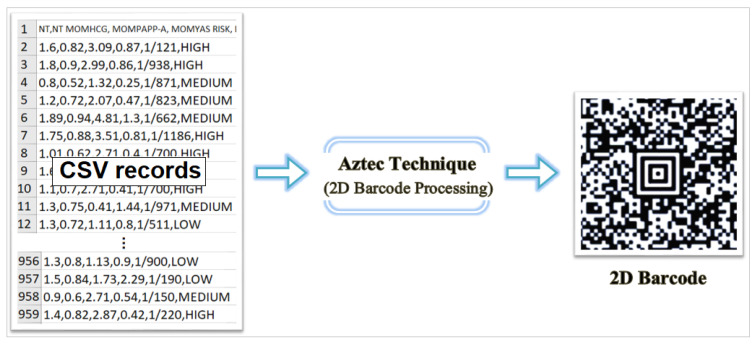
Application of the Aztec barcode technique to raw numerical data from the original dataset. The resulting 2D Aztec code visually encodes structured input features (e.g., hCG-MoM, PAPP-AMoM, NT MoM, etc.) into a fixed-size image representation. This transformation enables the use of vision-based transformers for classification tasks in prenatal DS risk assessment.

**Figure 3:**
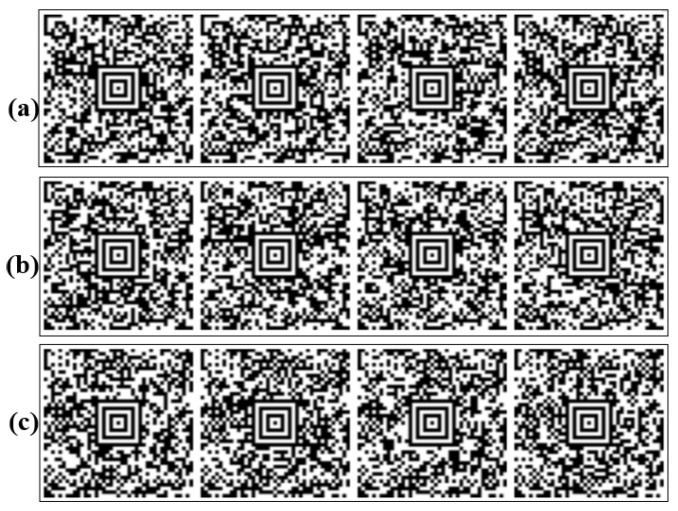
Sample Aztec-coded image set generated after pre-processing raw patient data. Each image corresponds to a different risk class used in the classification task: (**a**) high risk, (**b**) low risk, (**c**) medium risk. These visual representations are input to the vision-based transformer models for automated classification in the context of prenatal DS risk evaluation.

Barcode scanning refers to the technology of automatically reading data encoded through lines and shapes of varying thicknesses using optical scanning methods [28]. Traditional linear barcodes are categorized as one-dimensional (1D), while advanced formats, such as QR codes, are considered two-dimensional (2D). QR codes, including types such as QR, Data Matrix, and Aztec, have a higher data storage capacity because they encode information both horizontally and vertically. The Aztec code was used in this study. This technique uses a matrix barcode structure organized around a central finder pattern to generate 2D data. This spiral layout allows for more compact encoding of data without requiring a blank margin, making it more efficient than other 2D formats. Aztec codes also use the Reed–Solomon error correction algorithm, which can recover damaged data. Because of these advantages, they are commonly used in fields where data integrity and compact storage are critical, such as healthcare, logistics, and transportation [29,30]. In this study, patient data in CSV format were converted into Aztec barcode images as part of the preprocessing step. The pseudo-code provided in Algorithm 1 outlines the basic algorithm used for this transformation. Each row of the dataset was encoded into a unique Aztec barcode and saved as an image file for subsequent analysis. Figure 2 illustrates an example of how line data were transformed into Aztec barcodes, while Figure 3 shows sample images categorized by DS risk levels: high, medium, and low.

**Algorithm 1:** Pseudo-code of the Aztec technique
function csv_to_aztec_barcode (csv_file): *# Read CSV file*
  data = read_csv(csv_file)   aztec_barcodes = [] *# Generate AZTEC barcodes*
  for row in data:     barcode = create_aztec(row) *# Convert data to AZTEC barcode*
    save_image(barcode) *# Save 2D barcode*
  end

### 2.3. Transformer Models

The concept of Transformer was first developed in 2017 by researchers at the University of Toronto and Google and introduced in the paper “Attention is all you need” [31]. This study selected three different transformer-based models based on their complementary architectural structures and successful performance in visual classification tasks: Data-efficient Image Transformer v3 (DeiT3), Shifted Window Transformer (Swin), and Maximized Vision Transformer (MaxViT). DeiT3 was chosen because of its compact structure and effective performance in scenarios requiring limited data and computational power. This feature makes DeiT3 a suitable option for medical analysis tasks involving small datasets. The Swin transformer features a sliding window-based hierarchical architecture. This structure enables Swin transformer to effectively learn both local and global context, which is advantageous for medical barcode images that have variable structures but maintain a specific order. The MaxViT model combines convolutional layers and self-attention mechanisms within a grid-like structure to provide balanced representations of local details and global relationships. Using these three models together enables the extraction of versatile, rich features from data obtained by converting 1D clinical data into 2D Aztec barcode images. This integrative approach aims to enhance the system’s classification accuracy and overall robustness by leveraging the strengths of each model.

#### 2.3.1. DeiT3 Model

DeiT3, the third version of DeiT models, aims to achieve high performance with less data. It accurately classifies images in cases where data constraints exist. It is optimized for applications that require low data usage. Huge indicates that the model has a large parameter size. Patch14 means that the input image is divided into 14 × 14 patches, and 224 means that the model processes 224 × 224 images [32].

#### 2.3.2. MaxViT Model

MaxViT is a model that combines the strengths of Vision Transformers and convolutional neural networks (CNNs). It uses multi-scale attention mechanisms and performs well in visual tasks such as image classification, object detection, and segmentation. The model’s name indicates that it is a medium-sized model compatible with TensorFlow that processes images of size 224 × 224 [33].

#### 2.3.3. Swin Model

Swin efficiently processes local and global image features using a sliding attention mechanism within local windows. The model can perform multi-scale image analysis and is used in many areas, including image classification, object detection, and segmentation. Sliding windows provide better visual consistency. In the model, “Base” represents a medium-sized model; “patch4” indicates that the image is divided into 4 × 4 patches; “window7” indicates that 7 × 7 sliding windows are used; and “224” indicates that the model processes 224 × 224 images [34].

### 2.4. Feature Selection Methods: mRMR and RelieF

Feature selection is creating a subset of a feature vector according to specific criteria. It plays a vital role in reducing the data to be processed and removing unnecessary features [35]. Successful feature selection increases the learning performance of deep neural networks, reduces the learning time, and provides easy learning results. In this study, mRMR [36] and RelieF [37], which are supervised feature selection algorithms with high performance (Max-Relevance and Min-Redundancy), were used. Supervised feature selection algorithms are frequently used in classification problems. Its basic principle uses the relationship between features and labels. It aims to find the subset of the feature vector that achieves maximum classification performance for a given dataset.

The mRMR method selects features that are highly correlated with the target variable while minimizing redundancy among the selected features. This method typically evaluates the relationship between variables and the target using mutual information (MI). The basic mathematical expression of this approach is as follows:

(1) $$\underset{f_{i}\in S}{\max}\left[D\left(f_{i},\;C\right)-\;\frac{1}{\left|S\right|}\;\sum_{f_{i}\in \;S}R(f_{i},\;f_{j})\right]$$

The meanings of the parameters in Equation (1) are as follows: $f_{i}$ is a candidate feature; C is the class label. $D\left(f_{i},C\right)$ quantitatively expresses the information sharing between class labels $f_{i}$ and C. $R(f_{i},f_{j})$ indicates the amount of common information between features $f_{i}$ and $f_{j}$, and this value expresses the redundancy between them. S is defined as a subgroup consisting of previously determined characteristics. mRMR establishes a balance between maximum relationship and minimum repetition in order to select features that are highly informative but do not repeat each other. This ensures that the selected features are both meaningful and diverse [36].

RelieF is an example-based feature selection algorithm that evaluates the importance of each feature based on its ability to distinguish between similar examples from different classes. The algorithm determines the following two elements for each randomly selected example X: the closest neighbor example belonging to the same class, $X_{H}$ (closest match), and the closest neighbor belonging to a different class, $X_{M}$ (closest divergence).

(2) $$W\left[f\right]=W\left[f\right]-{diff\left(f,X,X_{H}\right)}^{2}+\;{diff(f,X,X_{M})}^{2}$$

This equation calculates the difference among the values of the f feature in the $diff\left(f,X_{i},X_{j}\right)$, $X_{i}$, and $X_{j}$ samples; this distance is usually measured using the absolute value or square difference method. Features that can distinguish more strongly between classes are given higher weights and are therefore evaluated more meaningfully [37].

The reasons for selecting the two methods in this study are as follows: mRMR was chosen because it is successful at identifying features that are highly correlated with the target variable and do not overlap. Relief was selected because it excels at capturing local feature interactions. Together, these methods provide an effective, complementary solution that maintains classification accuracy during dimension reduction.

### 2.5. Proposed Approach

FTS involves tests designed to evaluate the risk of fetal chromosomal abnormalities, such as DS. Although traditional methods estimate risk levels based on these results, they often overlook important factors. This study proposes a hybrid approach to overcome these limitations and provide accurate risk predictions using FTS data.

The proposed method integrates advanced artificial intelligence techniques and consists of five main stages: data preprocessing, transformer model training, feature extraction, feature fusion, feature selection, and classification.

**Figure 4:**
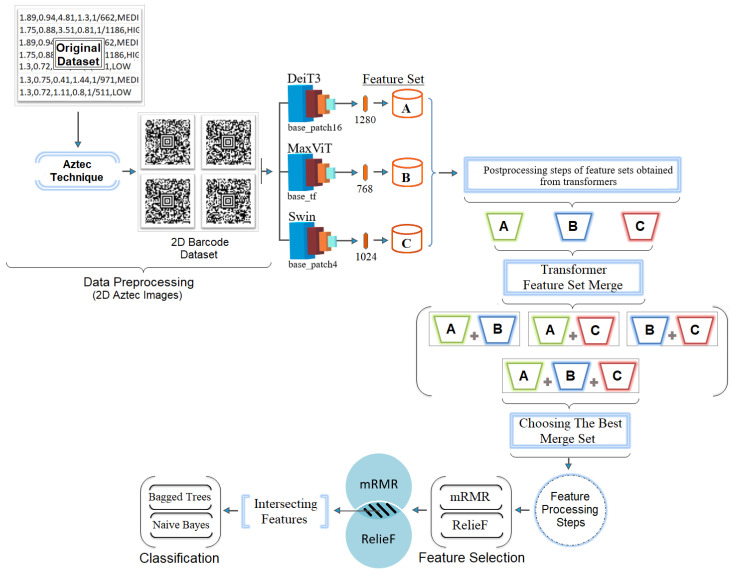
Model design of hybrid approach proposed for this study.

In the preprocessing stage, 1D patient data stored in CSV format is transformed into 2D barcode images using the Aztec encoding technique. This transformation enables the use of 2D transformer models, which are more efficient at extracting features compared to their 1D counterparts. In the second stage, the Aztec-based images are trained using three transformer models: DeiT3, MaxViT, and Swin. These models were chosen for their relatively simple architecture and lower computational demands. In the third stage, feature sets are extracted from the final layers of each model: DeiT3 (1280 × number of images), MaxViT (768 × number of images), and Swin (1024 × number of images). The fourth stage involves merging feature sets from different models and evaluating their classification performance. Various combinations were tested (e.g., DeiT3 and Swin, DeiT3 and MaxViT, MaxViT and Swin, and all three combined). Machine learning algorithms, including Bagged Trees [38] and Naive Bayes [39], were used to assess the performance of each merged feature set. The combination of all three models (DeiT3, MaxViT, and Swin) yielded the best performance and was selected for further processing. In the final stage, feature selection techniques—mRMR and RelieF—were applied to reduce dimensionality while maintaining classification performance. Each method was tested with varying numbers of selected features (750, 1000, 1250). The most effective feature subsets from both selection methods were identified, and their intersection was used to train new models. This step aimed to enhance performance while reducing computational and hardware costs, ultimately improving the efficiency and practicality of the proposed system. The overall workflow of the proposed hybrid AI system is illustrated in Figure 4.

### 2.6. Experimental Setup and Performance Evaluation

#### 2.6.1. Software and Hardware Environment

Transformer model training and data processing steps were performed in the Jupyter Notebook (7.0.8) environment. This tool enables transparent and repeatable workflows by allowing code to be executed in chunks, visualizations to be presented interactively, and errors to be identified and resolved immediately [40]. Thanks to these features, both model development processes and data preparation tasks have become more accessible and sustainable in clinical research. Subsequent processes, including feature fusion, feature selection, intersection, and classification, were conducted using MATLAB 2023. MATLAB was selected for its robust machine learning and signal processing toolboxes, which facilitated the efficient implementation of feature analysis techniques such as mRMR and RelieF.

All analyses were conducted on a personal computer equipped with an Intel Core i7 processor (3.40 GHz), 32 GB RAM, and a dedicated GPU with 10 GB memory (NVIDIA Corporation, Santa Clara, CA, USA), ensuring smooth handling of computationally intensive tasks. The system was assembled using components sourced from ASUS (Taipei, Taiwan) and Kingston Technology (Fountain Valley, CA, USA).

#### 2.6.2. Performance Metrics

The confusion matrix is a widely used tool in classification tasks [41], and it was employed in this study to evaluate the accuracy of the proposed models. Performance metrics derived from the confusion matrix were calculated using the equations provided in Equations (3)–(7) [39,41]. These equations involve key terms such as positive (P), negative (N), true (T), and false (F). The corresponding performance metrics include accuracy (Acc), F-score (F-Scr), specificity (Sp), sensitivity (Se), and precision (Pre). Among these, accuracy is frequently used in measurement evaluations and performs well with balanced datasets. The F-score, on the other hand, is particularly valuable in situations where class imbalance is present [42,43,44].

(3) $$\mathrm{Acc}=\;\frac{TP+TN}{TP+TN+FP+FN}$$

(4) $$F-\mathrm{Scr}=\;\frac{2\times TP}{2\times TP+FP+FN}$$

(5) $$\mathrm{Sp}=\;\frac{TN}{TN+FP}$$

(6) $$\mathrm{Se}=\;\frac{TP}{TP+FN}$$

(7) $$\mathrm{Pre}=\;\frac{TP}{TP+FP}$$

#### 2.6.3. Model Parameters and Configuration

Table 2 provides detailed information on the preferred parameters and their values for the transformer models and machine learning methods used in this study. The preferred parameters for the machine learning methods are the default values. These values were used as the default setting in all steps of the experimental analyses. Five-fold cross-validation was applied in the final experimental steps to evaluate the generalizability and robustness of the proposed model. The dataset was randomly partitioned into five subsets of equal size. Each subset was used once as a validation fold while the remaining four formed the training set. This process was repeated five times, and the resulting metrics were averaged to provide a comprehensive evaluation of performance stability.

## 3. Results

The experimental analysis of this study consisted of five steps. In the first step of the experimental analysis, data preprocessing was performed. In this step, each patient record was converted into a 2D Aztec-based barcode image. The primary aim of this transformation was to enable training with two-dimensional models, as 2D CNNs and transformer architectures are capable of deeper learning and more effective feature extraction than their one-dimensional counterparts. This preprocessing step laid the groundwork for the second step, where transformer-based training was conducted.

**Figure 5:**
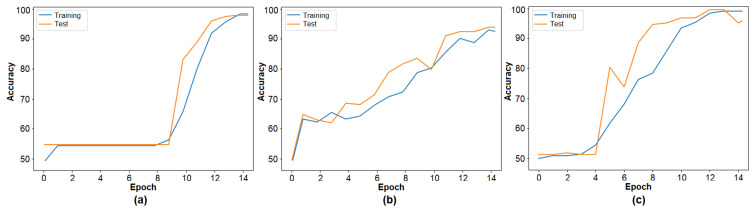
Classification performances of transformers: (**a**) Deit3, (**b**) MaxViT, (**c**) Swin.

The second step of the experimental analysis involved training the barcode image set using three transformer models. The training accuracy graphs are presented in Figure 5, and the performance results are summarized in Table 3. DeiT3 achieved the highest accuracy at 98.26%, followed by Swin with 96.88% and MaxViT with 94.79%. Among the three, DeiT3 demonstrated superior performance under identical conditions. The high classification accuracy across all three models supports the effectiveness of the barcode-based data representation and validates the model selection for the proposed hybrid approach. In the subsequent step, the feature sets were extracted from the final layer of each transformer model for further processing. The number of features derived from each model is detailed in Table 3.

The third step of the experiment conducted for this study involved combining feature sets. The feature sets obtained from three transformers were merged (DeiT3 and MaxViT, DeiT3 and Swin, MaxViT and Swin, DeiT3 and MaxViT and Swin). In total, four merged feature sets were obtained. Information about the number of features of the merged sets is given in Table 4.

**Figure 6:**
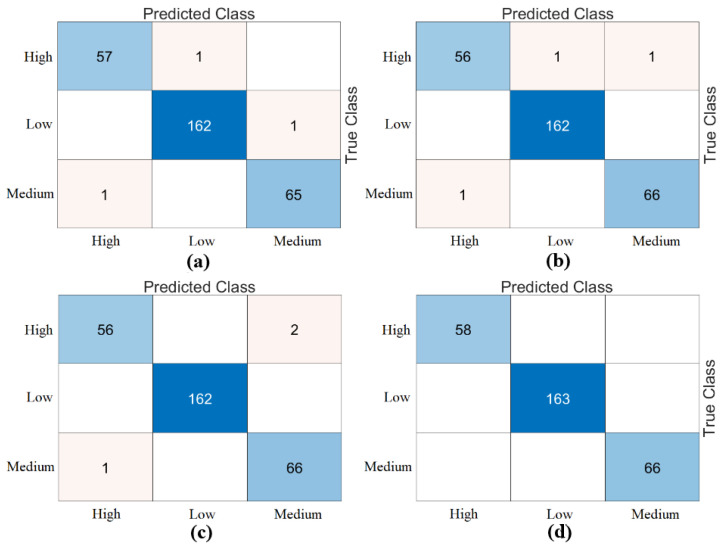
Confusion matrices for combined feature sets using Bagged Trees: (**a**) DeiT3–MaxViT (W), (**b**) DeiT3–Swin (V), (**c**) MaxViT–Swin (Y), (**d**) DeiT3–MaxViT–Swin (Z).

**Figure 7:**
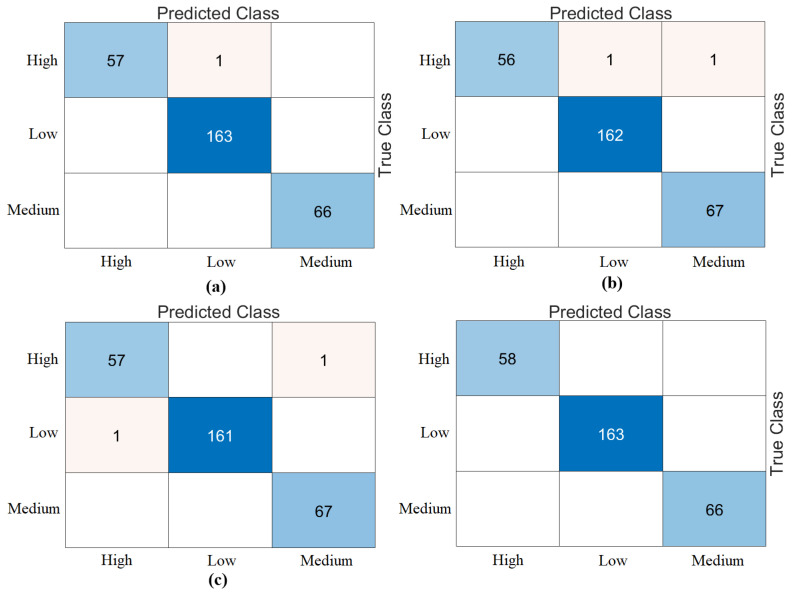
Confusion matrices obtained by combining the feature sets between models (classifier: Naïve Bayes): (**a**) DeiT3 and MaxViT (W), (**b**) DeiT3 and Swin (V), (**c**) MaxViT and Swin (Y), (**d**) DeiT3 and MaxViT and Swin (Z).

**Figure 8:**
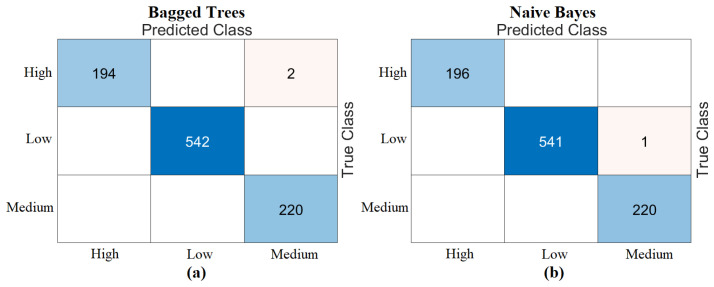
Confusion matrices of best performing feature set “DeiT3 & MaxViT & Swin” analyzed by cross-validation (k = 5 selected): (**a**) Bagged Trees, (**b**) Naïve Bayes.

The third step of the experimental analysis involved the use of Bagged Trees and Naive Bayes classifiers. Bagged Trees and Naive Bayes methods classified the combined feature sets. The confusion matrices obtained from the Bagged Trees analysis are shown in Figure 6. The metric results from the Bagged Trees analysis are given in Table 5. Similarly, the confusion matrices obtained from the Naive Bayes analysis are shown in Figure 7. The metric results from the analysis of this method are given in Table 6. Among the combined sets (W, V, Y, Z), the best performance result was obtained by the “DeiT3 & MaxViT & Swin” feature set represented by “Z”. The Bagged Trees and Naive Bayes methods preferred in the proposed approach achieved 100% overall accuracy. The performance results obtained on other feature sets also aligned with our expectations. In the analyses performed in this step, the Bagged Trees and Naive Bayes methods gave successful results and were compatible with the proposed approach. This step used a cross-validation technique to verify the success of the best-performing combined feature set “DeiT3, MaxViT, and Swin”. Using the cross-validation technique, a k-value of 5 was selected and reclassified using machine learning methods. The confusion matrices obtained by machine learning methods are shown in Figure 8. The metric results of the confusion matrices obtained with the cross-validation technique are given in Table 7. When analyzing Table 7, the Bagged Trees method achieved an overall accuracy of 99.79%, and the Naive Bayes method achieved an overall accuracy of 99.89%. The overall performance obtained with the cross-validation technique confirmed the previous analysis results. In the experimental analyses carried out so far, it was observed that the Naive Bayes method gives better results than the Bagged Trees method. In the next step, considering the time and hardware cost of the proposed approach, the classification process was continued using only the Naive Bayes method.

**Figure 9:**
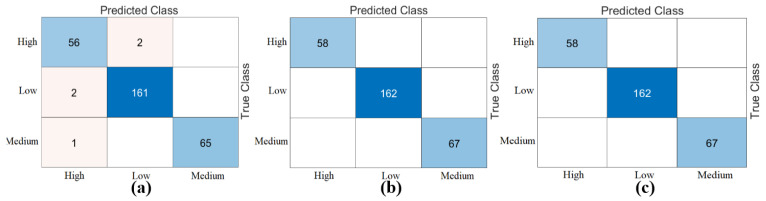
Naive Bayes confusion matrices of the “Z” dataset using the mRMR feature selection method (training/test rate: 0.7/0.3): (**a**) selection of the best 750 features, (**b**) selection of the best 1000 features, (**c**) selection of the best 1250 features.

**Figure 10:**
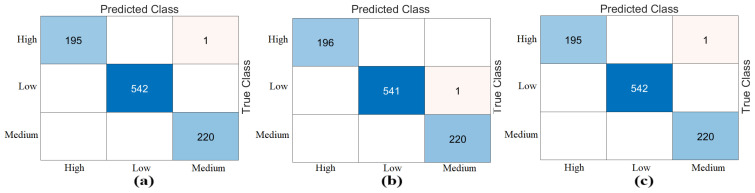
Naive Bayes confusion matrices of the “Z” dataset using the mRMR feature selection method (cross-validation, k = 5): (**a**) selection of the best 750 features, (**b**) selection of the best 1000 features, (**c**) selection of the best 1250 features.

In the fourth step, the mRMR and RelieF methods were used as feature selection algorithms. In this step, the operations were performed on the “Z” dataset, which showed the best performance in the previous step. The total number of feature columns in the “Z” dataset was 3072. In this step, feature selection was performed to determine the best 750, best 1000, and best 1250 features among 3072 features. The training data was then classified using the Naive Bayes method, which included 70% of the training data and 30% of the test data. The cross-validation technique was also used to validate the analyses performed on the training data (k = 5 was chosen). The feature columns and score rankings selected by the mRMR and RelieF methods are presented in Supplementary Materials, Table S1. The “Feature Selection” tool in the MATLAB 2023 interface was used for the feature selection algorithms. The best 750 features, 1000 features, and 1250 features were selected from the “Z” dataset using the mRMR method. These feature sets were then classified as train/test data using the Naive Bayes method. As a result of the classification, the best 1000 features and 1250 feature sets gave 100% overall accuracy. The best 750 features gave an overall accuracy of 98.25%. The confusion matrices of this analysis are shown in Figure 9. The feature sets (750, 1000, 1250) obtained with mRMR were then reclassified with Naive Bayes using cross-validation. An overall accuracy of 99.89% was obtained for all feature sets. The confusion matrices of these classification results are shown in Figure 10.

**Figure 11:**
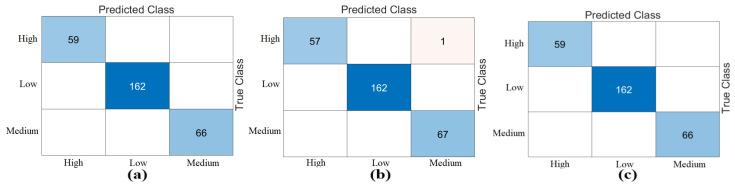
Naive Bayes confusion matrices of the “Z” dataset using the RelieF feature selection method (training/test rate: 0.7/0.3): (**a**) selection of the best 750 features, (**b**) selection of the best 1000 features, (**c**) selection of the best 1250 features.

**Figure 12:**
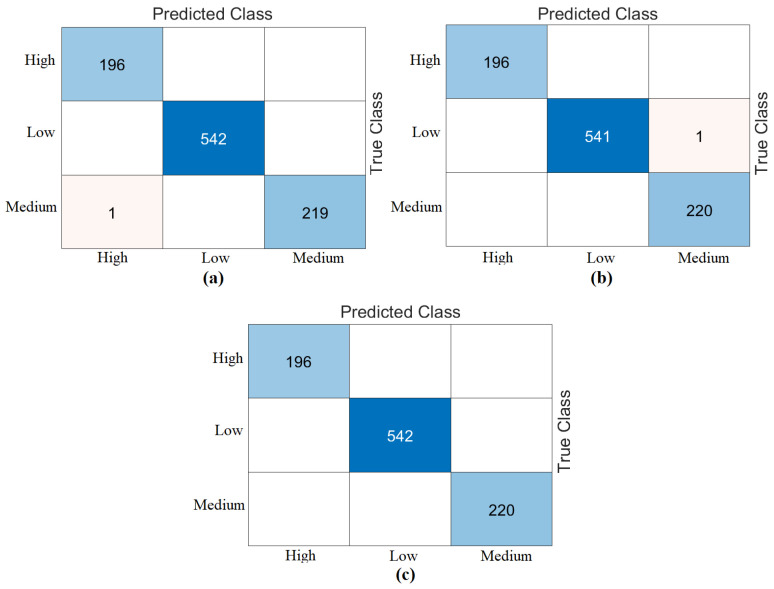
Naive Bayes confusion matrices of the “Z” dataset using the RelieF feature selection method (cross-validation, k = 5): (**a**) selection of the best 750 features, (**b**) selection of the best 1000 features, (**c**) selection of the best 1250 features.

Continuing with the analysis in the fourth step, the best 750 features, 1000 features, and 1250 features were selected from the “Z” dataset using the RelieF method. These feature sets were then classified as train/test data using the Naive Bayes method. As a result of the classification, the best 750 features and 1250 feature sets gave 100% overall accuracy. The best 1000 features gave an overall accuracy of 99.65%. The confusion matrices of this analysis are shown in Figure 11. The feature sets (750, 1000, 1250) obtained with RelieF were then reclassified with Naive Bayes using cross-validation. As a result of the classification, the best 750 and the best 1000 feature sets gave an overall accuracy of 99.89%. The best 1250 feature set achieved an overall accuracy of 100%. The confusion matrices of these classification results are shown in Figure 12. Table 8 shows the overall accuracy of the analyses (training/testing, cross-validation) performed with two feature selection methods (mRMR, RelieF) considering the confusion matrices. When analyzing Table 8, the best performance is the 1250 feature set (with the RelieF method), which gives 100% overall accuracy success considering both the training/test data and the cross-validation technique. The RelieF method gave more effective results than the mRMR method.

**Figure 13:**
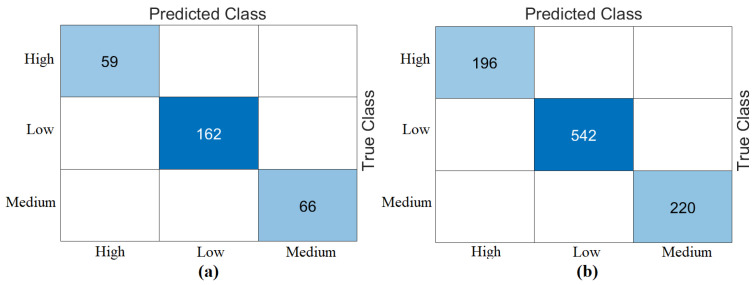
Confusion matrices from Naive Bayes classification using 527 intersecting features from the top 1250 mRMR and RelieF selections: (**a**) train/test split 0.7/0.3, (**b**) 5-fold cross-validation.

In the last step of the experimental analyses (step 5), the feature intersection approach was used. The aim was to identify common feature columns between the best feature columns selected by the two feature methods (mRMR, RelieF). Since the best performance in the fourth step was based on the first 1250 feature sets, the common/intersecting feature columns between the best 1250 features were identified in this step. In other words, the intersecting feature columns of the best set of 1250 feature columns obtained from the “Z” set by the mRMR method and the best set of 1250 feature columns obtained from the “Z” set by the RelieF method were determined, and a new feature set containing a total of 527 feature columns was created. This feature set (column set of 527 features) was processed and classified using the Naive Bayes method in both train/test and cross-validation formats. As a result of the classification, 100% overall accuracy was obtained from both the training/testing and cross-validation data. The confusion matrices obtained from the analysis of this step are shown in Figure 13. The results of the analysis are given at the end of Table 8. In the last step of the proposed approach, it was observed that the feature set containing 527 feature columns performed better.

**Figure 14:**
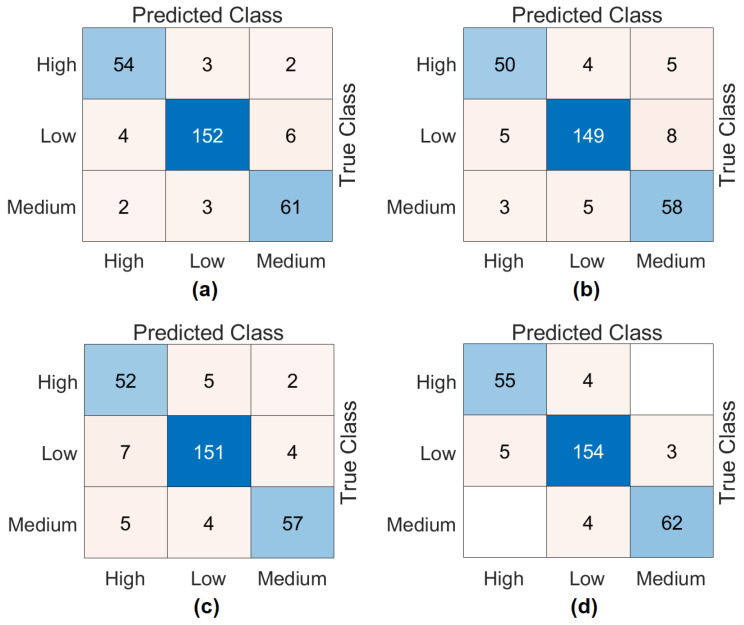
Confusion matrices obtained by training deep learning models using the Aztec-based image set obtained as a result of the experimental analysis of this study (training/test: 0.7/0.3); (**a**) AlexNet, (**b**) GoogLeNet, (**c**) ResNet-18, (**d**) VGG-16.

An additional experiment was conducted to compare the proposed transformer-based framework with conventional deep learning models. For this evaluation, Aztec barcode images generated from 1D prenatal screening data were used to train four widely adopted CNN architectures: AlexNet, GoogLeNet, ResNet-18, and VGG-16. These models were then tested on the same dataset, and the resulting confusion matrices are shown in Figure 14. The classification accuracies obtained were 93.03% with AlexNet, 89.54% with GoogLeNet, 90.59% with ResNet-18, and 94.42% with VGG-16. The metric results obtained from the analyses are given in Table 9. While these CNN models demonstrated satisfactory performance, the proposed hybrid transformer-based framework produced superior results, especially in terms of classification accuracy. These results highlight the effectiveness of transformer architectures in extracting discriminative features from barcode-encoded clinical data and affirm the Aztec transformation method’s ability to improve model performance across various deep learning paradigms.

The experimental analysis of the proposed approach in this study consists of five steps, each of which contributes to the performance of the previous step. The cross-validation technique was also used in the analyses to maintain the validity of the proposed approach. The experimental analyses of this study showed that the proposed approach successfully detects the patient’s risk level based on the FTS data. Although the proposed model achieved 100% accuracy in the experimental dataset, this result may not generalize to broader, more diverse populations. Further validation on larger, heterogeneous datasets is necessary to confirm the robustness of the model. This represents a major limitation of the current study.

The proposed hybrid approach consists of the following five key steps: (1) Data preprocessing, in which 1D patient data is converted into 2D Aztec barcode images to enable effective input into 2D transformer models. (2) Transformer model training, in which DeiT3, MaxViT and Swin models are trained on the barcode dataset. (3) Feature extraction and fusion, in which features from the final layers of the models are extracted and combined to form multiple merged feature sets. (4) Feature selection: mRMR and RelieF algorithms are applied to identify the most relevant features, including an intersection set of 527 features. (5) Classification: the refined feature sets are evaluated using machine learning classifiers (particularly Naive Bayes) to achieve high accuracy and efficiency. This multi-stage design provides DS risk estimation with better performance.

## 4. Discussion

FTS tests are essential for assessing the risk of genetic diseases like DS and other chromosomal disorders in the fetus. In this study, a hybrid AI approach was developed to predict fetal risk levels based on first trimester screening data. The classification results achieved with transformer-based models (e.g., DeiT3: 98.26% accuracy) and selected features demonstrate that the approach can provide high predictive accuracy using a limited number of biomarkers. Early diagnosis, along with timely and appropriate clinical interventions, significantly impacts fetal health. While FTS test accuracy is critical, low sensitivity and false negatives can cause unnecessary anxiety and treatment errors. Improving model accuracy enhances early and precise disease diagnosis. However, several challenges remain for widespread clinical application. The proposed approach developed in this study combines transformer models and advanced data processing techniques to accurately determine fetal risk levels from FTS data. The results showed that this methodology provides high accuracy and efficiency.

Though this study is technical in scope, its findings have significant clinical implications. The proposed AI framework is designed for future clinical applications, especially as a decision support system for first trimester prenatal screenings. Due to its efficient computational design and reduced feature complexity, the model can be integrated with electronic medical records (EMRs) to enable real-time risk evaluation. This would help healthcare providers make more consistent decisions, especially in under-resourced settings. As a proof-of-concept study, future research will focus on clinical validation, usability testing, and practical deployment scenarios in collaboration with obstetric clinics. The developed hybrid AI method contributes to early diagnosis and intervention processes by determining DS risk more accurately and quickly in the first trimester screening. The model’s Aztec barcode conversion and transformer-based analysis capabilities can be incorporated into the patient data of clinical laboratories and doctors’ decision support systems. This aims to deliver significant time and cost savings in prenatal screening processes.

Table 10 presents a comparison of the proposed approach with similar studies. The studies in Table 10 are analyses of 1D data.

Pi et al. [45] compared six machine learning methods that could predict high-risk pregnancies using data collected in Bangladesh. According to their findings, the MLP method produced the most accurate results, achieving an accuracy rate of 91%. Togunwa et al. [46] developed a comprehensive hybrid model that combines ANN and RF methods for classifying risks to maternal health that may arise during pregnancy. The model demonstrated high performance, achieving 95% accuracy on test data. Mutlu et al. [47] evaluated six machine learning algorithms for identifying health threats to mothers with high-risk pregnancies in their study. The DT method achieved the highest accuracy rate of 89.16%. Jamel et al. [48] developed a model that combines PCA with a stacked community classifier method to predict risks to maternal health. This model, which uses attributes obtained through PCA, demonstrated high accuracy with a rate of 98.25%. Saleh et al. [49] proposed a deep learning framework supported by the IoMT and XAI to reduce maternal mortality. The system continuously monitors pregnant individuals’ vital signs, evaluates risk factors based on the collected data, and provides regional-level risk predictions with 92.6% accuracy.

As shown in Table 10, previous studies relied on traditional machine learning and preprocessing methods. In contrast, our study paved the way for training 2D transformer models by converting data into barcode images and extracting more efficient features. We combined the feature sets obtained from the models with feature selection and intersection in the final processing step. The proposed approach in this study has achieved a classification accuracy of up to 100% under certain experimental conditions.

This study has several limitations. Firstly, the dataset is relatively small. Secondly, the model was only tested with specific AI approaches. Nevertheless, applying mRMR and RelieF—powerful feature selection techniques—alongside analyzing different transformer-based models has significantly mitigated the impact of these limitations.

Future studies will aim to enhance the model’s generalizability by developing it using larger and more diverse datasets. In addition to expanding the dataset, future research could focus on integrating longitudinal data from repeated screenings, exploring ensemble learning methods, and evaluating model performance using multicenter, cross-regional data sources. These steps would strengthen the model’s robustness and clinical adaptability. Additionally, future studies should consider incorporating maternal age as a key predictive variable given its well-established association with the risk of having a child with DS. Stratified modeling based on maternal age groups could improve accuracy and clinical relevance.

## 5. Conclusions

This study presents a hybrid AI approach designed to improve the accuracy, efficiency, and reliability of DS risk prediction during the first trimester of prenatal screening. The method involves converting one-dimensional patient data, including clinically relevant biomarkers such as nuchal translucency, hCG, and PAPP-A, into 2D Aztec barcode representations. This enables more effective feature extraction through transformer-based models such as DeiT3, MaxViT, and Swin.

The results show that combining the proposed pipeline with robust feature selection methods such as mRMR and RelieF can achieve very high classification accuracy. Specifically, integrating 1250 features selected via RelieF and 527 intersecting features from mRMR produced the most successful classification outcome. This method provides promising results in predicting the risk of congenital anomalies and can reduce unnecessary amniocentesis referrals, minimizing stress for patients. The proposed approach offers several advantages, including improved diagnostic accuracy, workload reduction, efficient resource use, and clinical scalability. It demonstrates the potential of AI in healthcare, setting a new standard for FTS data evaluation. Future research could extend this approach to other prenatal screening tests and larger clinical settings.

Although the proposed approach yielded promising results, it is important to note that the findings are based on a limited, ethically approved, private dataset. As such, this study should be considered proof-of-concept. This proof-of-concept study lays the groundwork for future clinical integration of AI-based risk assessment tools in prenatal care, with planned collaborations for real-world validation and usability testing. Future research will focus on validating the proposed method using larger, multicenter, and more diverse datasets to ensure broader clinical applicability and generalizability. Future improvements to the proposed method may include integrating maternal age and other demographic factors to improve predictive performance and ensure greater applicability to diverse populations.

## Figures and Tables

**Table 1:** The attributes explanation of the collected dataset.

Name	Explanation	Data Type	Range (Min–Max)	Mean	Std Dev
hCG-MoM	Human Chorionic Gonadotropin (hCG), Method of Medians (MoM). hCG is a hormone secreted by the placenta during pregnancy and is found in high levels in the early stages. The MoM value is obtained by dividing the measured value by the average value during the gestational week.	Numeric	0.35–4.81	1.64	0.74
PAPP-AMoM	Pregnancy-Associated Plasma Protein A (PAPP-A). PAPP-A, known as pregnancy-associated plasma protein A, is produced by the placenta during pregnancy.	Numeric	0.18–127	1.20	5.78
NT	Nuchal Translucency (NT). This is the measurement of the nuchal translucency of the fetus by ultrasound.	Numeric	0.45–4.50	1.48	0.52
NT MoM	It is obtained by dividing NT by the mean value during gestational weeks.	Numeric	0.12–4	0.99	0.49
Down Risk	DS is a risk classification. Below 1:250 is considered high risk (High), between 1:250 and 1:1000 is considered medium risk (Medium), and above 1:1000 is considered low risk (Low).	Categorical			

**Table 2:** Parameters and values of transformer models and other methods used in proposed approach.

Model/Method	Parameter	Preference/Value
DeiT3, MaxViT, Swin	Classifier	Linear
	Epoch	15
	Learning rate	10^−4^
	Loss function	Cross Entropy
	Mini-batch	4
	Optimization	SGD
	Training and testing rate	70–30%
Bagged Trees	Learning rate	0.1
	Number of learners	30
	Number of predictors to sample	All
	Maximum number of splits	670
Naïve Bayes	Misclassification costs	Default
	Preset	Gaussian

**Table 3:** Test data metric results of transformers (%).

Transformer	Features	Se	Pre	F-Scr	Acc
Deit3	1280	98.26	98.27	98.24	98.26
MaxViT	768	94.79	95.20	94.63	94.79
Swin	1024	96.88	97.27	96.82	96.88

Se: sensitivity; Pre: precision; F-Scr: F1-score; Acc: accuracy.

**Table 4:** The number of features of the models combined using the feature sets and the letter symbols they represent.

Representing Letter Symbol	Models with Combined Features	Feature Counts
W	DeiT3 and MaxViT	2048
V	DeiT3 and Swin	2304
Y	MaxViT and Swin	1792
Z	DeiT3 and MaxViT and Swin	3072

**Table 5:** Metric results of confusion matrices obtained from analysis of model-based fused feature sets. (classifier: Bagged Trees; training/test rate: 0.7:0.3).

Feature Merging Between Models	Class	Se	Spe	Pre	F-Scr	Acc
W	High	98.27	99.56	98.27	98.27	98.95
	Low	99.38	99.18	99.38	99.38	
	Medium	98.48	99.54	98.48	98.48	
V	High	96.55	99.56	98.24	97.39	98.95
	Low	100	99.18	99.38	99.69	
	Medium	98.50	99.54	98.50	98.50	
Y	High	96.55	99.56	98.24	97.39	98.95
	Low	100	100	100	100	
	Medium	98.50	99.09	97.05	97.77	
Z	High	100	100	100	100	100
	Low	100	100	100	100	
	Medium	100	100	100	100	

**Table 6:** Metric results of confusion matrices obtained from analysis of model-based fused feature sets. (Classifier: Naïve Bayes; training/test rate: 0.7:0.3).

Feature Merging Between Models	Class	Se	Spe	Pre	F-Scr	Acc
W	High	98.27	100	100	99.13	99.65
	Low	100	99.19	99.39	99.69	
	Medium	100	100	100	100	
V	High	96.55	100	100	98.24	99.30
	Low	100	99.19	99.38	99.69	
	Medium	100	99.54	98.52	99.25	
Y	High	98.27	99.56	98.27	98.27	99.30
	Low	99.38	100	100	99.69	
	Medium	100	99.54	98.52	99.25	
Z	High	100	100	100	100	100
	Low	100	100	100	100	
	Medium	100	100	100	100	

**Table 7:** Metric results of the confusion matrices obtained from the analysis of the best performing feature set “Z”. (Cross-validation rate/k = 5 was chosen).

Feature Merging Between Models	Classifier	Class	Se	Spe	Pre	F-Scr	Acc
**Z ** ‘DeiT3 & MaxViT & Swin’	Bagged Trees	High	98.97	100	100	99.48	99.79
		Low	100	100	100	100	
		Medium	100	99.72	99.09	99.54	
	Naïve Bayes	High	100	100	100	100	99.89
		Low	99.81	100	100	99.90	
		Medium	100	99.86	99.54	99.77	

**Table 8:** Overall accuracy of mRMR and RelieF for classification of selected features (%).

Feature Set	ML	Feature Selection	Top Features	Acc (Test:0.3)	Acc (Cross Val./k = 5)
‘Z’ 3072 features	Naïve Bayes	mRMR	750	98.25	99.89
			1000	100	99.89
			1250	100	99.89
		RelieF	750	100	99.89
			1000	99.65	99.89
			1250	100	100
mRMR top 1250 features in “Z” and RelieF top 1250 features in “Z”		mRMR and RelieF intersection	527 *	100	100

* Number of common/intersecting feature columns among top 1250 feature columns determined by mRMR and RelieF.

**Table 9:** Analysis results (%) of confusion matrices obtained from training deep learning models on Aztec-based image set.

Model	Dataset	Class	Se	Spe	Pre	F-Scr	Acc
AlexNet	Aztec-based image set	High	91.53	97.26	90	90.76	93.03
		Low	93.83	95.04	96.20	95	
		Medium	92.42	96.26	88.41	90.37	
GoogLeNet		High	84.75	96.28	86.21	85.47	89.54
		Low	91.98	92.31	94.30	93.12	
		Medium	87.88	93.87	81.69	84.67	
ResNet-18		High	88.14	94.55	81.25	84.55	90.59
		Low	93.21	92.37	94.38	93.79	
		Medium	86.36	97.13	90.48	88.37	
VGG-16		High	93.22	97.74	91.67	92.44	94.42
		Low	95.06	93.60	95.06	95.06	
		Medium	93.94	98.58	95.38	94.66	

**Table 10:** Comparison with similar studies in literature.

Article	Year	Model/Method	Acc. (%)
Pi et al. [45]	2025	Multilayer perceptron (MLP)	91
Togunwa et al. [46]	2023	Random forest (RF), ANN	95
Mutlu et al. [47]	2023	Decision tree (DT)	89.16
Jamel et al. [48]	2024	Principal component analysis (PCA), ML	98.25
Saleh et al. [49]	2024	Internet of medical things (IoMT), Explainable artificial intelligence (XAI)	92.60
This paper	2025	Barcode technique, transformers, Feature fusion and intersection	100

## Data Availability

All data generated or analyzed during this study are available for sharing when appropriate request is directed to the corresponding author. The implementation code used in this study was made publicly available to ensure transparency and reproducibility. Due to ethical concerns, the original patient dataset cannot be shared. However, the code can be accessed at the following GitHub repository: https://github.com/mtogacar/DS_Syndrome_Risk_Prediction- (accessed on 3 June 2025).

## Supplementary Materials

The following supporting information can be downloaded at: https://www.mdpi.com/article/10.3390/diagnostics15121444/s1, Table S1: Feature Selection List.

## References

[1] Charlotte T.N., Aurore N.D., Charlotte B., Esther B., Eugene B.P. (2015). Prenatal Diagnosis of Congenital Malformations in Douala General Hospital. Open J. Obstet. Gynecol..

[2] Kiani A.K., Paolacci S., Scanzano P., Michelini S., Capodicasa N., D’agruma L., Notarangelo A., Tonini G., Piccinelli D., Farshid K.R. (2020). Prenatal Genetic Diagnosis: Fetal Therapy as a Possible Solution to a Positive Test. Acta Biomed..

[3] Benn P. (2002). Combined Second-Trimester Biochemical and Ultrasound Screening for Down Syndrome. Obstet. Gynecol..

[4] De Biasio P., Siccardi M., Volpe G., Famularo L., Santi F., Canini S. (1999). First-Trimester Screening for Down Syndrome Using Nuchal Translucency Measurement with Free β-HCG and PAPP-A between 10 and 13 Weeks of Pregnancy—The Combined Test. Prenat. Diagn..

[5] Bahado-Singh R.O., Oz A.U., Gomez K., Hunter D., Copel J., Baumgarten A., Mahoney M.J. (2000). Combined Ultrasound Biometry, Serum Markers and Age for Down Syndrome Risk Estimation. Ultrasound Obstet. Gynecol..

[6] Cuckle H. (2000). Biochemical Screening for Down Syndrome. Eur. J. Obstet. Gynecol. Reprod. Biol..

[7] Orlandi F., Damiani G., Hallaban T.W., Krantz D.A., Macri J.N. (1997). First-trimester Screening for Fetal Aneuploidy: Biochemistry and Nuchal Translucency. Ultrasound Obstet. Gynecol..

[8] Malone F.D., Ball R.H., Nyberg D.A., Comstock C.H., Saade G.R., Berkowitz R.L., Gross S.J., Dugoff L., Craigo S.D., Timor-Tritsch I.E. (2005). First-Trimester Septated Cystic Hygroma: Prevalence, Natural History, and Pediatric Outcome. Obstet. Gynecol..

[9] Burtis C.A., Bruns D.E. (2014). Tietz Fundamentals of Clinical Chemistry and Molecular Diagnostics-E-Book: Tietz Fundamentals of Clinical Chemistry and Molecular Diagnostics.

[10] Tsakmaki E., Ververi A., Chatzakis C., Cavoretto P., Sotiriadis A. (2024). Genomic Abnormalities in Apparently Isolated Polyhydramnios and the Role of Confirmed Fetal Phenotype: A Systematic Review and Meta-Analysis. Am. J. Obstet. Gynecol. MFM.

[11] Muñoz-Cortes M., Arigita M., Falguera G., Seres A., Guix D., Baldrich E., Acera A., Torrent A., Rodriguez-Veret A., Lopez-Quesada E. (2012). Contingent Screening for Down Syndrome Completed in the First Trimester: A Multicenter Study. Ultrasound Obstet. Gynecol..

[12] Yeganegi M., Danaei M., Azizi S., Jayervand F., Bahrami R., Dastgheib S.A., Rashnavadi H., Masoudi A., Shiri A., Aghili K. (2025). Research Advancements in the Use of Artificial Intelligence for Prenatal Diagnosis of Neural Tube Defects. Front. Pediatr..

[13] He F., Lin B., Mou K., Jin L., Liu J. (2021). A Machine Learning Model for the Prediction of down Syndrome in Second Trimester Antenatal Screening. Clin. Chim. Acta.

[14] Nix B., Wright D., Baker A. (2007). The Impact of Bias in MoM Values on Patient Risk and Screening Performance for Down Syndrome. Prenat. Diagn..

[15] Huang T., Meschino W.S., Okun N., Dennis A., Hoffman B., Lepage N., Rashid S., Aul R., Farrell S.A. (2013). The Impact of Maternal Weight Discrepancies on Prenatal Screening Results for Down Syndrome. Prenat. Diagn..

[16] Neocleous A.C., Nicolaides K.H., Schizas C.N. (2016). First Trimester Noninvasive Prenatal Diagnosis: A Computational Intelligence Approach. IEEE J. Biomed. Health Inform..

[17] Koivu A., Korpimäki T., Kivelä P., Pahikkala T., Sairanen M. (2018). Evaluation of Machine Learning Algorithms for Improved Risk Assessment for Down’s Syndrome. Comput. Biol. Med..

[18] Yang J., Ding X., Zhu W. (2018). Improving the Calling of Non-Invasive Prenatal Testing on 13-/18-/21-Trisomy by Support Vector Machine Discrimination. PLoS ONE.

[19] Alonso E., Beristain A., Burgos J., Gurrutxaga I. (2025). Comparison of Machine Learning Algorithms to Predict Down Syndrome During the Screening of the First Trimester of Pregnancy. Appl. Sci..

[20] Durmuşoğlu A., Ay M.M., Unutmaz Durmuşoğlu Z.D. (2020). A Classification Model for Predicting Fetus with down Syndrome—A Study from Turkey. Appl. Artif. Intell..

[21] Uzun O., Kaya H. Prenatal Risk Assessment of Down’s Syndrome by Probabilistic Classifiers. Proceedings of the Signal Processing and Communications Applications Conference (SIU).

[22] Catic A., Gurbeta L., Kurtovic-Kozaric A., Mehmedbasic S., Badnjevic A. (2018). Application of Neural Networks for Classification of Patau, Edwards, Down, Turner and Klinefelter Syndrome Based on First Trimester Maternal Serum Screening Data, Ultrasonographic Findings and Patient Demographics. BMC Med. Genom..

[23] Wøjdemann K.R., Shalmi A.C., Christiansen M., Larsen S.O., Sundberg K., Brocks V., Bang J., Nørgaard-Pedersen B., Tabor A. (2005). Improved First-trimester Down Syndrome Screening Performance by Lowering the False-positive Rate: A Prospective Study of 9941 Low-risk Women. Ultrasound Obstet. Gynecol..

[24] Nguyen-Hoang L., Sahota D.S., Pooh R.K., Duan H., Chaiyasit N., Sekizawa A., Shaw S.W., Seshadri S., Choolani M., Yapan P. (2024). Validation of the First-trimester Machine Learning Model for Predicting Pre-eclampsia in an Asian Population. Int. J. Gynecol. Obstet..

[25] Verma D., Agrawal S., Iwendi C., Sharma B., Bhatia S., Basheer S. (2022). A Novel Framework for Abnormal Risk Classification over Fetal Nuchal Translucency Using Adaptive Stochastic Gradient Descent Algorithm. Diagnostics.

[26] Chen Y.-T., Chen G., Lin Y.-S. (2025). Machine Learning-Based Prediction of First Trimester Down Syndrome Risk in East Asian Populations. Risk Manag. Healthc. Policy.

[27] Ramanathan S., Sangeetha M., Talwai S., Natarajan S. (2018). Probabilistic Determination Of Down’s Syndrome Using Machine Learning Techniques. Proceedings of the 2018 International Conference on Advances in Computing, Communications and Informatics (ICACCI).

[28] Mulac A., Mathiesen L., Taxis K., Gerd Granås A. (2021). Barcode Medication Administration Technology Use in Hospital Practice: A Mixed-Methods Observational Study of Policy Deviations. BMJ Qual. Saf..

[29] Hutchinson S.A., Shadid J.N., Tuminaro R.S. (1995). Aztec User’s Guide, Version 1.0.

[30] Aztec Code. https://en.wikipedia.org/wiki/Aztec_Code.

[31] Zhang Y., Liu C., Liu M., Liu T., Lin H., Huang C.-B., Ning L. (2023). Attention Is All You Need: Utilizing Attention in AI-Enabled Drug Discovery. Brief. Bioinform..

[32] Paçal İ., Kunduracıoğlu İ. (2024). Data-Efficient Vision Transformer Models for Robust Classification of Sugarcane. J. Soft Comput. Decis. Anal..

[33] Lee S., Koo K., Lee J.H., Lee G., Jeong S., Seongjun O., Kim H. (2024). Vision Transformer Models for Mobile/Edge Devices: A Survey. Multimed. Syst..

[34] Agar M., Aydin S., Cakmak M., Koc M., Togacar M. (2024). Detection of Thymoma Disease Using MRMR Feature Selection and Transformer Models. Diagnostics.

[35] Abed R.Q., Dikmen M., Aydemir E., Barua P.D., Dogan S., Tuncer T., Palmer E.E., Ciaccio E.J., Acharya U.R. (2023). Automated Reading Level Classification Model Based on Improved Orbital Pattern. Multimed. Tools Appl..

[36] Hanchuan Peng, Fuhui Long, Ding, C (2005). Feature Selection Based on Mutual Information Criteria of Max-Dependency, Max-Relevance, and Min-Redundancy. IEEE Trans. Pattern Anal. Mach. Intell..

[37] Urbanowicz R.J., Meeker M., La Cava W., Olson R.S., Moore J.H. (2018). Relief-Based Feature Selection: Introduction and Review. J. Biomed. Inform..

[38] Dudeja D., Noonia A., Lavanya S., Sharma V., Kumar V., Rehan S., Ramkumar R. (2023). Breast Cancer Diagnosis Using Bagging Decision Trees with Improved Feature Selection. Proceedings of the RAiSE-2023.

[39] Peretz O., Koren M., Koren O. (2024). Naive Bayes Classifier—An Ensemble Procedure for Recall and Precision Enrichment. Eng. Appl. Artif. Intell..

[40] Ochkov V.F., Stevens A., Tikhonov A.I. (2022). Jupyter Notebook, JupyterLab—Integrated Environment for STEM Education. Proceedings of the 2022 VI International Conference on Information Technologies in Engineering Education (Inforino).

[41] Božić D., Runje B., Lisjak D., Kolar D. (2023). Metrics Related to Confusion Matrix as Tools for Conformity Assessment Decisions. Appl. Sci..

[42] Çalışkan A. (2023). Detecting Human Activity Types from 3D Posture Data Using Deep Learning Models. Biomed. Signal Process. Control.

[43] Yildirim M., Cengil E., Eroglu Y., Cinar A. (2023). Detection and Classification of Glioma, Meningioma, Pituitary Tumor, and Normal in Brain Magnetic Resonance Imaging Using Deep Learning-Based Hybrid Model. Iran J. Comput. Sci..

[44] Alaca Y., Basaran E., Celik Y. (2024). Enhancing Anomaly Detection in Large-Scale Log Data Using Machine Learning: A Comparative Study of SVM and KNN Algorithms with HDFS Dataset. ADBA Comput. Sci..

[45] Pi X., Wang J., Chu L., Zhang G., Zhang W. (2025). Prediction of High-Risk Pregnancy Based on Machine Learning Algorithms. Sci. Rep..

[46] Togunwa T.O., Babatunde A.O., Abdullah K.-R. (2023). Deep Hybrid Model for Maternal Health Risk Classification in Pregnancy: Synergy of ANN and Random Forest. Front. Artif. Intell..

[47] Mutlu H.B., Durmaz F., Yücel N., Cengil E., Yıldırım M. (2023). Prediction of Maternal Health Risk with Traditional Machine Learning Methods. NATURENGS MTU J. Eng. Nat. Sci. Mal. Turgut Ozal Univ..

[48] Jamel L., Umer M., Saidani O., Alabduallah B., Alsubai S., Ishmanov F., Kim T., Ashraf I. (2024). Improving Prediction of Maternal Health Risks Using PCA Features and TreeNet Model. PeerJ Comput. Sci..

[49] Saleh S.N., Elagamy M.N., Saleh Y.N.M., Osman R.A. (2024). An Explainable Deep Learning-Enhanced IoMT Model for Effective Monitoring and Reduction of Maternal Mortality Risks. Futur. Internet.

